# Comparing endometrial preparation protocols and clinical outcomes: lessons learned from 3507 vitrified-warmed euploid transfers

**DOI:** 10.1007/s10815-026-03910-5

**Published:** 2026-05-19

**Authors:** Erika Pittana, Alessandro Ruffa, Giosuè Giordano Incognito, Federica Battista, Federica Innocenti, Marilena Taggi, Michele Morelli, Maurizio Guido, Carla Ettore, Giuseppe Rizzo, Laura Rienzi, Filippo Maria Ubaldi, Danilo Cimadomo, Alberto Vaiarelli

**Affiliations:** 1 Genera, Clinica Valle Giulia, IVIRMA Global Research Alliance, Roma, Italy; 2 Department of Biomedicine and Prevention, Tor Vergata University, Roma, Italy; 3 Obstetrics and gynecology unit, Maternal Child Department, ARNAS Garibaldi Nesima, Catania, Italy; 4 Department of Biology and Biotechnology “Lazzaro Spallanzani”, University of Pavia, Pavia, Italy; 5 IVIRMA Italia, IVIRMA Global Research Alliance, Roma, Italy; 6 Department of Pharmacy and Health Sciences and Nutrition, Università della Calabria, Cosenza, Italy; 7 Department of Maternal and Child Health and Urological Sciences, Sapienza University of Rome, Rome, Italy; 8 Department of Biomolecular Sciences, University of Urbino, Urbino, Italy; 9 Department of Pharmacy and Health Sciences and Nutrition, Università della Calabria, Cosenza, Italy; 10 Department of Biology and Biotechnology “Lazzaro Spallanzani”, University of Pavia, Pavia, Italy; 11 IVIRMA Italia, IVIRMA Global Research Alliance, Roma, Italy

**Keywords:** Vitrified-warmed embryo transfer, PGT-A, Euploid, Endometrial preparation, Modified natural cycle, Hormonal replacement therapy

## Abstract

**Purpose:**

To evaluate whether artificial endometrial preparation using hormone replacement therapy (HRT) influences clinical and neonatal outcomes compared with modified natural cycles (mNC) in single euploid vitrified–warmed blastocyst transfers.

**Methods:**

This retrospective cohort study analyzed single euploid vitrified–warmed blastocyst transfers performed at a single IVF center over an 11-year period. Endometrial preparation was conducted either through HRT or mNC. Clinical and neonatal outcomes were compared between groups. Statistical analyses included multivariable logistic regression models, adjusted for relevant confounders, with generalized estimating equations to account for repeated embryo transfers (ETs) within patients.

**Results:**

A total of 3507 ETs were conducted in 2257 patients. Mean age was 37.5 ± 3.2 years at oocyte pick-up and 37.8 ± 3.3 years at ET. HRT was associated with a higher miscarriage rate (*N* = 167/1034, 16.2% vs *N* = 70/700, 10%; OR 1.72, 95% CI 1.27–2.33) and a lower live birth rate (*N* = 867/2129, 40.7% vs *N* = 630/1378, 45.7%; OR 0.83, 95% CI 0.72–0.96) than mNC. Additionally, HRT was associated with a higher prevalence of large for gestational age newborns.

**Conclusions:**

mNC endometrial preparation may show some clinical benefit compared to HRT. The latter remains necessary in specific cases, such as hypothalamic amenorrhea (excluded in this study), but mNC protocols should be preferred whenever possible. Randomized controlled trials in Preimplantation Genetic Testing for Aneuploidy (PGT-A) cycles are essential to confirm these findings.

## Introduction

Vitrified–warmed embryo transfer (ET) has increased globally, becoming the predominant approach in ART. In Europe, 365,111 vitrified–warmed ETs were performed in 2021, representing approximately 37% of all ETs, compared with 2010 when they accounted for less than 30% [1, 2]. This rise reflects both technical advances in vitrification and a shift in clinical practice. Vitrified–warmed ET is often considered safer than fresh ET, particularly to prevent ovarian hyperstimulation syndrome (OHSS) in high-responders and is necessary in Preimplantation Genetic Testing (PGT) cycles [3]. Meta-analyses and large cohorts show equal or higher live birth rates after vitrified–warmed ET versus fresh ET in normo-ovulatory women [3, 4], supporting the use of freeze-all strategies to improve the endometrial environment and reduce supraphysiologic exposure to oestradiol observed in stimulated cycles [5]. Concurrently, there has been a global move toward single ET (SET), which has become the standard of care to minimize the risk of multiple pregnancy. In PGT-A cycles, transferring one euploid blastocyst provides implantation rates of 50–60% per transfer, even in advanced maternal age, while drastically reducing twin-related complications [6, 7]. By removing embryo chromosomal abnormalities as a major confounder, euploid SET has also allowed a less biased investigation of non-embryonic factors, particularly endometrial ones, involved in implantation. Nevertheless, residual embryo-related influences, including morphological and developmental features, may still affect implantation potential [8]. In light of the widespread and steadily increasing adoption of vitrified–warmed ET, the endometrial preparation method has assumed a pivotal role in determining vitrified–warmed ET success, attracting growing scientific interest. The three main approaches are: natural cycle (NC), modified NC (mNC), and hormone replacement therapy (HRT). Each protocol has distinct advantages and drawbacks. HRT offers higher flexibility in scheduling and reduced monitoring burden, facilitating planning for both patients and clinics. However, the absence of a corpus luteum (CL) and its vasoactive factors, such as relaxin and vascular endothelial growth factor, may influence placentation [9]. Reduced relaxin levels have been linked to increased systemic vascular resistance and impaired maternal adaptation to pregnancy, predisposing to hypertensive disorders and abnormal placentation [9], True NC preserves more physiologic dynamics but is less predictable and requires more frequent ultrasound (US) and/or hormonal monitoring. mNC differs from true NC by including pharmacological ovulation triggering, which provides partial scheduling control while maintaining CL physiology [10]. Recent studies, including randomized control trial evidence, report higher first-trimester bleeding and numerically increased preeclampsia risk in artificial cycles, as well as greater susceptibility to hypertensive and vascular maladaptation even when analyses are restricted to euploid vitrified–warmed ET [11, 12]. Evidence also indicates that endometrial protocol choice may modulate maternal vascular and endocrine adaptations affecting neonatal outcomes [13]. Nevertheless, the question remains unresolved, as current evidence regarding the impact of endometrial preparation on outcomes following vitrified-warmed ET is still limited. To date, only a relatively small number of studies have specifically investigated endometrial preparation strategies in the setting of vitrified–warmed euploid blastocyst transfer [11, 14–18]. Available data are scarce and heterogeneous, often including different embryo stages, variable transfer policies, or focusing predominantly on clinical outcomes, while neonatal data remain largely underreported. To address these gaps, the present study was designed to evaluate the impact of endometrial preparation protocols, specifically mNC and HRT, in a large private setting, focusing exclusively on single euploid blastocyst transfers and integrating clinical and neonatal outcomes.

## Materials and methods

### Study population

This retrospective cohort study included all patients who underwent vitrified-warmed single euploid blastocyst transfer at a private IVF clinic in Rome (IVIRMA Global Research Alliance, Genera, Clinica Valle Giulia), between January 2012 and December 2023, after ICSI cycles with their own eggs. Women were assigned to different endometrial preparation protocols mainly based on logistical considerations, such as the preference of HRT whenever remote follow-up was needed because of geographical distance from the center. Endocrine ovulatory causes of female infertility were excluded to avoid the putative bias introduced by irregular or anovulatory menstrual cycles. Ethical committee and data protection approvals were obtained for the retrospective analysis of pseudonymized data to investigate the efficacy and efficiency of IVF protocols and strategies.

### IVF procedures

Ovarian stimulation (OS) was conducted with GnRH antagonist, GnRH agonist protocols, or with progestin primed approach, with the choice of regimen tailored to individual patients’ characteristics and ovarian reserve parameters. Ovulation was triggered with either GnRH agonist or hCG, according to patients’ characteristics and based on gynecologists’ evaluations [19, 20]. Oocytes were retrieved 35–36 h after trigger, and only ICSI was conducted as previously detailed [21, 22]. All cycles involved embryo culture in a controlled humidified atmosphere (37 °C, 6% CO2 and 5% O2) until full blastocyst expansion on day 5–7, when the embryos were submitted to trophectoderm biopsy for PGT-A according to a protocol not involving day 3 zona pellucida drilling [23, 24]. Comprehensive chromosome testing was conducted at an external laboratory. Only full chromosome uniform aneuploidies were diagnosed. Blastocyst morphology was graded according to Gardner’s score based on expansion, inner cell mass (ICM), and trophectoderm quality [25]. All patients underwent screening for thyroid function before ET. Hysteroscopy was performed only when an irregular endometrium was detected at US. Only single euploid blastocysts transfer(s) were performed 2 h after warming as previously described [23, 24, 26]. Endometrial preparation was conducted with either a mNC or HRT protocol. In both protocols, blastocyst transfer was run under US guidance using a soft catheter (Cook Soft-Pass, Cook Medical, Ireland), as previously described [27].

#### Modified natural cycle (mNC)

In the mNC protocol, the growth of the dominant follicle and endometrial development were monitored from the early follicular phase by transvaginal US. When the leading follicle reached an average diameter of 16–18 mm, and the endometrium displayed a trilaminar pattern with a thickness ≥ 7 mm, ovulation was triggered with a single subcutaneous injection of 10.000 IU urinary hCG (Gonasi; IBSA, Switzerland). Luteal phase support was initiated 36–40 h after the trigger and consisted of vaginal micronized progesterone at a total dose of 600 mg/day, administered in divided doses (Progeffik; Effik, Italy). In cases of confirmed pregnancy, supplementation continued until at least 10 weeks of gestation. ET was scheduled at hCG + 7 days or Progesterone + 5 days, according to the developmental stage of the blastocyst, to ensure synchronization with the endometrial secretory phase.

#### Hormone replacement therapy (HRT)

In the HRT protocol, endometrial preparation was initiated in the early follicular phase with oral estradiol valerate 6 mg/day (Progynova; Bayer, Germany), administered in three daily doses starting on the second or third day of the menstrual cycle and titrated according to US assessments of endometrial thickness and pattern. Once the endometrium reached a trilaminar appearance with a thickness ≥ 7 mm, progesterone supplementation was initiated with vaginal micronized progesterone 800 mg/day (Progeffik; Effik, Italy), defining day 0 of the luteal phase. Blastocyst transfer was scheduled on the fifth day of progesterone exposure to align with physiological implantation timing. Estradiol and progesterone administration continued until at least 10 weeks of gestation in ongoing pregnancies.

### Data collection

Baseline characteristics collected through chart review for each patient included maternal age, body mass index (BMI), female infertility etiology, maternal age at oocyte pick-up (OPU), OS duration, number of days from menstruation to OS initiation and to OPU, the OS protocol employed, the type of ovulation trigger used, the endometrial preparation protocol adopted (mNC or HRT), and blastocyst Gardner’s score, and day of trophectoderm biopsy (5, 6, or 7). Clinical outcomes included positive pregnancy test per ET, biochemical pregnancy loss per positive pregnancy test, miscarriage per clinical pregnancy, and live birth per ET. Biochemical pregnancy loss was defined as a transient rise in serum β-hCG levels (≥ 50 IU/L) measured 11 days after ET, in at least two measurements and increasing, but not associated with any US evidence of intrauterine or ectopic pregnancy. Clinical miscarriage was defined as the spontaneous loss of a clinically recognized intrauterine pregnancy before 22 weeks of gestation. Live birth was defined as the delivery of a viable infant beyond 22 weeks of gestation [28]. Gestational age and birthweight data were collected by telephone interviews, but 10.2% of the deliveries were lost to follow-up (*N* = 154/1506). Preterm birth was defined as delivery before 37 gestational weeks, and late-term birth as delivery at or beyond 41 weeks [29, 30]. Birthweight percentiles were calculated using the INTERGROWTH-21st international standards, defining small-for-gestational-age (SGA) as < 10th percentile and large-for-gestational-age (LGA) as > 90th percentile for gestational age [31].

### Statistical analysis

Continuous variables were reported as mean ± standard deviation (SD) or as median with first (Q1) and third (Q3) quartiles, and the Shapiro–Wilk test was adopted to confirm the Gaussian distribution of the data. The Mann–Whitney *U* or Student’s *t*-tests were adopted to outline significant differences among each comparison. The chi-square test was instead adopted for categorical variables that were expressed as *n*/*N* and percentages. Logistic regressions were conducted to confirm significant associations. Associations between baseline characteristics and outcomes were evaluated using univariate logistic regression analyses, and variables with significant associations were included in multivariate models. A Generalized Estimating Equation (GEE) approach was applied to account for multiple ETs performed within the same patient. A sub-analysis was also conducted only among the first ET from each patient. All statistical tests were two-sided, and a *p*-value < 0.05 was considered statistically significant. All statistical analyses were performed with the software SPSS v30.0 (IBM, Armonk, NY, USA).

## Results

A total of 2257 patients were included (mean maternal age at OPU and at ET: 37.5 ± 3.2 years and 37.8 ± 3.3 years, respectively; mean BMI: 22.1 ± 3.2 kg/m^2^). The causes of female infertility were advanced maternal age (71%), endometriosis (6.4%), tubal factor (8.9%) and idiopathic (14%). Overall, 2479 OPU procedures were performed. A GnRH antagonist stimulation protocol was used in 93.2% of the cases, GnRH agonist in 3.1% and progestin-primed ovarian stimulation in 3.7%. Agonist trigger was used in 67.9% of the cases, and hCG in 32.1%. OS lasted on average 10.8 ± 1.7 days. Days between menstruation and OS were 2.9 ± 1.1 and days between menstruation and OPU were 13.6 ± 1.8. Most patients underwent a single OPU and ET, with a mean of 1.4 OPUs per patient and 1.6 ETs per ET. A total of 3507 vitrified-warmed single euploid blastocyst transfers were analyzed: 1378 following mNC endometrial preparation and 2129 after HRT. Mean maternal age at OPU and ET and BMI were comparable between groups. Although some statistically significant differences were observed in the distribution of blastocyst day at trophectoderm biopsy and Gardner’s score (Table 1), these differences were relatively small and unlikely to be clinically meaningful.

**Table 1:** Comparison of embryo transfer (ET) cycle characteristics between modified natural cycle (mNC) and hormonal replacement therapy (HRT) protocols

ET characteristic	mNC *N* = 1378	HRT *N* = 2129	*p*-value
**Maternal age at OPU (**years) (mean ± SD)	37.1 ± 3.3	37.3 ± 3.2	0.133
**Maternal age at ET (**years) (mean ± SD)	37.7 ± 3.4	38.0 ± 3.2	0.063
**BMI** (mean ± SD)	22.1 ± 3.1	22.1 ± 3.2	0.985
**Main cause of female infertility**			0.090
Idiopathic *N* (%)	236 (17.1%)	324 (15.2%)	
Endometriosis *N* (%)	82 (6%)	155 (7.3%)	
Tubal factor *N* (%)	139 (10.1%)	184 (8.6%)	
Advanced maternal age *N* (%)	921 (66.8%)	1466 (68.9%)	
**Specific ET**			0.465
First *N* (%)	913 (66.3%)	1344 (63.1%)	
Second *N* (%)	311 (22.6%)	521 (24.5%)	
Third *N* (%)	107 (7.8%)	182 (8.5%)	
Fourth or more *N* (%)	47 (3.4%)	82 (3.8%)	
**Day of trophectoderm biopsy**			0.010
5 *N* (%)	660 (47.9%)	913 (42.9%)	
6 *N* (%)	644 (46.7%)	1075 (50.5%)	
7 *N* (%)	74 (5.4%)	141 (6.6%)	
**Gardner’s score**			0.030
AA *N* (%)	934 (68.0%)	1422 (66.8%)	
AB, BA *N* (%)	136 (9.8%)	266 (12.5%)	
BB, AC, CA *N* (%)	135 (9.6%)	167 (7.8%)	
CC, BC, CB *N* (%)	173 (12.6%)	274 (12.9%)	

*BMI *body mass index, *OPU *oocyte pick up

Positive pregnancy test rates per ET were similar between the endometrial preparation protocols (Table 2). Similarly biochemical pregnancy loss rates per positive pregnancy test did not differ (Table 2). Conversely, miscarriage rates per clinical pregnancy were significantly lower in mNC cycles compared with HRT cycles, even when adjusting for maternal age at ET, day of trophectoderm biopsy and Gardner’s score in a multivariate logistic regression analysis with a GEE approach (multivariate OR: 1.72, 95% CI 1.27–2.33, *p* < 0.001) (Table 2). Consequently, live birth rates per ET were also significantly higher in mNC cycles, even after adjusting for the same variables (multivariate OR 0.83, 95% CI 0.72–0.96, *p* = 0.012) (Table 2). The data were also confirmed among only the first ETs conducted from each patient (Table 2).

**Table 2:** Comparison of clinical outcomes per single euploid blastocyst transfer between modified natural cycles (mNC) and hormone replacement therapy (HRT) endometrial preparation among all ETs and only first ETs from each patient

Outcome of all ETs	mNC	HRT	Multivariate OR HRT vs mNC*, 95% CI, *p*-value
Positive pregnancy test rate per ET *n*/*N* (%)	780/1378 (56.6%)	1184/2129 (55.6%)	-
Biochemical pregnancy loss rate per positive pregnancy test *n*/*N* (%)	80/780 (10.3%)	150/1184 (12.7%)	-
Miscarriage rate per clinical pregnancy *n*/*N* (%)	70/700 (10.0%)	167/1034 (16.2%)	1.72, 95% CI 1.27–2.33, *p* < 0.001
Live birth rate per ET *n*/*N* (%)	630/1378 (45.7%)	867/2129 (40.7%)	0.83, 95% CI 0.72–0.96, *p* = 0.012
**Outcome of first ETs**	**mNC**	**HRT**	Multivariate OR HRT vs mNC*, 95% CI, *p*-value
Positive pregnancy test rate per ET *n*/*N* (%)	543/913 (59.5%)	778/1344 (57.9%)	-
Biochemical pregnancy loss rate per positive pregnancy test *n*/*N* (%)	59/543 (10.9%)	92/778 (11.8%)	-
Miscarriage rate per clinical pregnancy *n*/*N* (%)	52/484 (10.7%)	109/686 (15.9%)	1.50, 95% CI 1.05–2.14, *p* = 0.026
Live birth rate per ET *n*/*N* (%)	432/913 (47.3%)	577/1344 (42.9%)	0.84, 95% CI 0.71*–*0.99, *p* = 0.050

^*^Adjusted for maternal age at ET, day of trophectoderm biopsy, and Gardner’s score

**Fig. 1:**
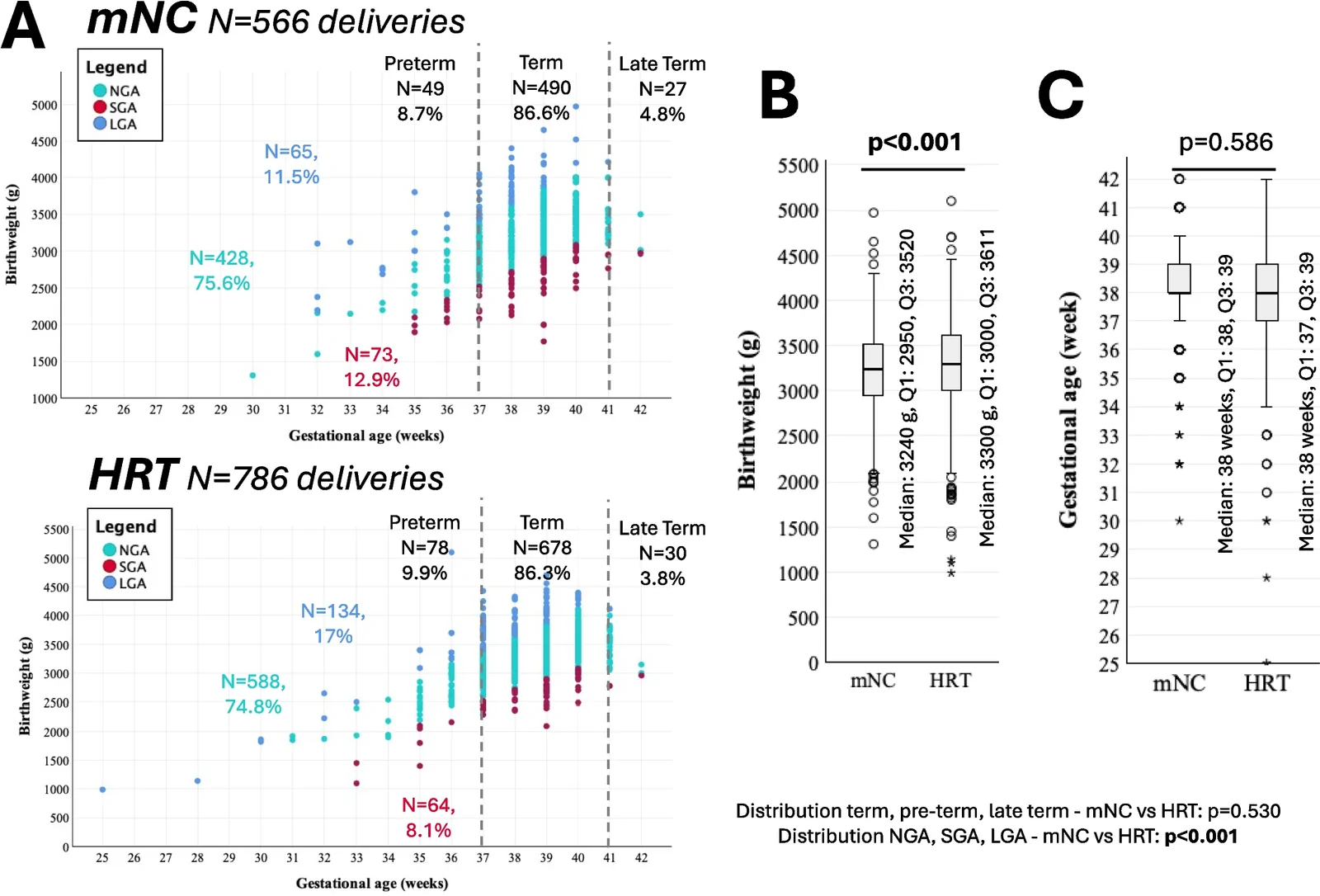
Neonatal outcomes after modified natural cycle (mNC) or hormonal replacement therapy (HRT) for endometrial preparation. **A** The dispersion plot for the association between birthweight and gestational age at delivery in the two groups. The dotted gray lines represent the thresholds for pre-term and late-term delivery. Normal for gestational age (NGA) newborns are shown as green dots, small for gestational age (SGA) as red dots, and large for gestational age (LGA) as blue dots. All *p*-values for these comparisons are shown at the bottom of the figure. **B** The boxplots for the birthweight of the newborns in the two groups. **C** The boxplots for the gestational age at delivery in the two groups

Neonatal outcomes were obtained by telephone interviews for 566 deliveries after mNC and 786 after HRT. The two groups showed similar prevalence of normal for gestational age newborns, but lower prevalence of LGA and higher prevalence of SGA were reported in the mNC group (*p* < 0.001) (Fig. 1A). In fact, the birthweight was significantly higher after HRT (*p* < 0.001), while the gestational age was similar in the two groups (Fig. 1B, C). This association was confirmed also when adjusted for maternal BMI (HRT vs mNC: + 72 g, 95%CI + 1 to + 147 g, *p* = 0.050). The rate of twin deliveries was also the same in the study groups (mNC: *N* = 6/566, 1.1% vs HRT: *N* = 9/786, 1.1%; *p* = 0.999).

## Discussion

In this large retrospective cohort of vitrified-warmed single euploid blastocyst transfers performed in a large clinical setting, the association of endometrial preparation strategy with reproductive and neonatal outcomes was evaluated, comparing mNC with HRT. By restricting the analysis to single euploid ETs, this study aims to reduce embryo-related confounding and to allow for a less biased assessment of the maternal and endometrial contribution to implantation, pregnancy, and fetal growth. These results show that implantation success was comparable between mNC and HRT cycles. However, pregnancies conceived after HRT were characterized by a significantly higher miscarriage rate and, as a consequence, a lower live birth rate compared with mNC. These differences remained significant after applying statistical adjustments, including GEE models and correction for age at embryo transfer, day of trophectoderm biopsy, and Gardner’s score, and were confirmed in a sub-analysis restricted to first embryo transfers. Notably, this approach is supported by previous evidence indicating that subsequent embryo transfers should not be considered intrinsically less effective, as each transfer represents an independent event contributing to the cumulative live birth rate [32, 33]. Embryo implantation requires a receptive endometrium, a functional, normally developing embryo, and synchronized embryo-endometrial cross-talk, while the progression of pregnancy requires a coordinated systemic adaptation involving endocrine, immune, and vascular pathways [35]. In ovulatory cycles, these processes are orchestrated by the CL, which secretes a complex array of vasoactive and angiogenic mediators, that promote systemic vasodilation, endothelial remodelling, and early placental vascular development [36, 37]. Growing evidence indicates that pregnancies established in the absence of CL signalling, as occurs in artificial cycles, may exhibit impaired cardiovascular adaptation, including increased systemic vascular resistance, changes in uterine artery Doppler indices, and potentially suboptimal placental vascular remodeling, possibly reflecting the lack of luteal-derived vasoactive factors [34, 38, 39]. However, evidence is heterogeneous, with some studies reporting no significant difference [40]. These alterations may contribute to early decidual instability, microvascular dysfunction, and increased susceptibility to pregnancy loss. Our observation of a higher miscarriage rate in HRT cycles may be partly explained by some underlying mechanisms. Supraphysiologic estrogen levels may negatively affect placentation. However, similar hormonal conditions may also occur in fresh ET cycles, suggesting that estrogen exposure alone is unlikely to fully account for the observed differences. Instead, the endocrine environment in HRT cycles differs due to the absence of CL-derived factors and the lack of physiological cyclic hormonal fluctuations, which may influence both systemic and local endometrial responses. Moreover, in HRT cycles, the absence of the CL makes first-trimester pregnancies fully dependent on exogenous progesterone. Although luteal support is routinely provided, interindividual variability in absorption, metabolism, and tissue exposure may result in suboptimal endometrial progesterone levels in some cases [41]. Our findings contribute to a relatively limited body of evidence in the context of euploid ETs. Most available studies evaluated transfers of untested embryos, reporting either no significant differences between mNC and HRT protocols [42, 43] or outcomes similar to ours, with lower miscarriage rates and higher live birth rates associated with mNC [44]. When restricting the analysis to studies based on only euploid ETs, our results appear consistent with those of some recent retrospective investigations [12, 17]. Other retrospective studies, only one of which with a reasonably large sample size [14], did not demonstrate any difference in clinical outcomes between NC and HRT [18, 45]. A randomized study consistently reported similar implantation and clinical pregnancy rates between mNC and HRT, but lower live birth rates and higher pregnancy loss in HRT cycles [15]. Notably, a recent multicenter randomized control trial demonstrated a significant reduction in live birth rates following artificial preparation compared with mNC in ovulatory women undergoing single euploid blastocyst transfer, highlighting that vascular and placental maladaptation, rather than implantation failure, may underlie the adverse outcomes observed in HRT cycles. Our findings mirror this pattern in a large clinical cohort reflecting routine clinical practice within a PGT-based setting and extend it by integrating neonatal outcomes [11]. These additional data provide further insight on the potential downstream effects of endometrial preparation. Although gestational age, the proportion of term deliveries were similar between groups and the overall birthweight was just slightly different, more newborns conceived after mNC were SGA and less were LGA compared with those conceived after HRT, also after adjusting for maternal BMI. Such differences suggest that artificial hormonal environments may alter placental efficiency and fetal growth trajectories, possibly through sustained supraphysiologic exposure to estradiol and progesterone, the absence of physiological endocrine oscillation, and compensatory placental mechanisms which may promote increased nutrient transfer later in gestation [38, 41, 46, 47].

### Clinical implications and future perspectives

These results underscore the clinical relevance of endometrial preparation strategies in vitrified-warmed ET cycles. While HRT remains essential in specific clinical scenarios like anovulatory patients (excluded here), natural-based regimens may represent a preferable option whenever feasible. In this context, the Natural Proliferative Phase (NPP) protocol, has been recently proposed as an alternative approach in which vaginal progesterone is initiated in the presence of a proliferative endometrium ≥ 7 mm, without confirming ovulation, allowing preservation of endogenous follicular activity while maintaining scheduling flexibility [48]. Overall, additional studies are warranted to better elucidate the mechanisms underlying these differences and to refine patient selection, with the aim of optimizing both reproductive and perinatal outcomes.

### Strengths and limitations

A major strength of the present study is the strict methodological design, which focused exclusively on vitrified-warmed single euploid blastocyst transfers in patients without known endocrine ovulatory disorders. By controlling for embryo chromosomal constitution, day of trophectoderm biopsy and embryo quality, the analysis aimed to reduce embryo-related confounding and to provide a more focused evaluation of the potential impact of the endometrial preparation protocols on reproductive and neonatal outcomes. The exclusion of endocrine ovulatory causes of female infertility helped to avoid potential bias associated irregular or anovulatory menstrual cycles. The inclusion of neonatal endpoints further enhances the clinical relevance of the findings, enabling a comprehensive evaluation that extends beyond implantation. Another strength lies in the homogeneity of clinical management and laboratory procedures. All transfers were performed within a standardized setting, ensuring uniformity in embryo culture, vitrification and warming protocols, luteal phase support regimens, ET technique, and operators’ training. Nonetheless, some limitations must be acknowledged. The retrospective nature of the analysis and the non-random allocation of the patients in the two groups limit causal inference and leave room for residual confounding. Additionally, the lack of blood hormonal levels and biochemical or hemodynamic markers, such as circulating angiogenic factors (e.g., PlGF and PAPP-A) and uterine blood flow indices (e.g., pulsatility index), preclude a mechanistic correlation between clinical outcomes and underlying physiological processes. Another limitation is the absence of an evaluation of gestational outcomes, particularly considering evidence linking HRT cycles to increased rates of hypertensive disorders and preeclampsia [42, 43]. Finally, the single-center design may limit the generalizability of the results to broader populations with different demographic, metabolic, or clinical profiles.

## Conclusion

This study demonstrates that the type of endometrial preparation used in vitrified-warmed ET cycles can influence clinical and neonatal outcomes. While implantation was comparable between HRT and mNC, the former was associated with higher miscarriage rates and lower live birth rates. These findings suggest that artificial hormonal environments may not fully reproduce the physiological conditions necessary for sustained gestation. Furthermore, subtle differences observed in neonatal birthweight suggest that the effects of endometrial preparation may extend beyond conception, potentially influencing placental development and function. Further prospective multicenter studies are warranted to confirm these associations and elucidate the physiological pathways linking endometrial preparation to reproductive and neonatal outcomes, ultimately guiding a more precise and physiology-oriented tailoring of ART practice.

## Data Availability

The data underlying this article are available in the article and in its online supplementary material.
